# Supplementary material for: Longitudinal White Matter Microstructure and Transition to Psychosis in Individuals at Clinical High Risk
Source: bioRxiv. 2026 Sep 29:2026.09.27.754817. Preprint. [Version 1] doi: 10.64898/2026.09.27.754817 (PMC13648486; doi:10.64898/2026.09.27.754817)

Table S1

Hazard Ratios Consistently <1 Across All White Matter Tracts – No Hemisphere-Averaging

Tract	Baseline Model			Longitudinal Model		
	HR (95% CI)	*p*	p_{corr}	HR (95% CI)	*p*	p_{corr}
Global FA	0.64 (0.39 – 1.04)	.069	.202	0.51 (0.24 – 1.09)	.073	.158
Anterior Thalamic Radiation - Left	0.69 (0.43 – 1.11)	.115	.202	0.48 (0.24 – 0.98)	*.046	.158
Anterior Thalamic Radiation - Right	0.72 (0.45 – 1.14)	.144	.202	0.48 (0.23 – 0.98)	*.045	.158
Cingulum Cingulate Gyrus - Left	0.73 (0.45 – 1.18)	.181	.211	0.59 (0.29 – 1.18)	.112	.158
Cingulum Cingulate Gyrus - Right	0.66 (0.41 – 1.06)	.084	.202	0.68 (0.34 – 1.34)	.207	.217
Cingulum Hippocampus - Left	0.51 (0.29 – 0.89)	*.022	.202	0.51 (0.22 – 1.21)	.105	.158
Cingulum Hippocampus - Right	0.56 (0.33 – 0.95)	*.035	.202	0.70 (0.29 – 1.68)	.353	.353
Corticospinal Tract - Left	0.73 (0.46 – 1.17)	.173	.211	0.62 (0.30 – 1.30)	.164	.199
Corticospinal Tract - Right	0.68 (0.41 – 1.11)	.111	.202	0.61 (0.32 – 1.16)	.106	.158
Forceps Major	0.68 (0.43 – 1.09)	.100	.202	0.52 (0.27 – 1.00)	.051	.158
Forceps Minor	0.75 (0.47 – 1.20)	.215	.226	0.53 (0.28 – 1.00)	*.049	.158
Inferior Fronto Occipital Fasciculus - Left	0.72 (0.44 – 1.15)	.155	.204	0.55 (0.28 – 1.07)	.069	.158
Inferior Fronto Occipital Fasciculus - Right	0.67 (0.42 – 1.08)	.092	.202	0.51 (0.25 – 1.06)	.064	.158
Inferior Longitudinal Fasciculus - Left	0.69 (0.42 – 1.11)	.114	.202	0.60 (0.29 – 1.26)	.142	.186
Inferior Longitudinal Fasciculus - Right	0.64 (0.39 – 1.05)	.075	.202	0.50 (0.21 – 1.18)	.094	.158
Superior Longitudinal Fasciculus - Left	0.69 (0.43 – 1.12)	.125	.202	0.58 (0.29 – 1.16)	.102	.158
Superior Longitudinal Fasciculus - Right	0.69 (0.43 – 1.11)	.115	.202	0.64 (0.31 – 1.33)	.180	.199
Superior Longitudinal Fasciculus Temporal Part - Left	0.70 (0.43 – 1.13)	.135	.202	0.57 (0.27 – 1.20)	.113	.158
Superior Longitudinal Fasciculus Temporal Part - Right	0.75 (0.46 – 1.21)	.212	.226	0.63 (0.30 – 1.32)	.172	.199
Uncinate Fasciculus - Left	0.76 (0.47 – 1.21)	.226	.226	0.57 (0.29 – 1.12)	.086	.158
Uncinate Fasciculus - Right	0.71 (0.44 – 1.12)	.130	.202	0.55 (0.26 – 1.16)	.097	.158

Table S2

Influence of Interaction Terms of BLIPS and White Matter on Hazard Ratios for Transition to Psychosis Risk

Tract	Baseline Model			Longitudinal Model		
	HR (95% CI)	p	p_{corr}	HR (95% CI)	p	p_{corr}
Global FA	3.24 (0.59 – 17.76)	.158	.194	1.41 (0.07 – 30.17)	.764	.788
Anterior Thalamic Radiation	2.20 (0.40 – 12.13)	.334	.344	0.72 (0.08 – 6.34)	.685	.759
Cingulum Cingulate Gyrus	3.66 (0.73 – 18.30)	.105	.134	1.11 (0.08 – 15.04)	.919	.919
Cingulum Hippocampus	2.17 (0.42 – 11.24)	.327	.344	3.77 (0.47 – 30.56)	.149	.497
Corticospinal Tract	0.93 (0.23 – 3.82)	.916	.916	0.48 (0.05 – 4.24)	.396	.577
Forceps Major	5.86 (1.62 – 21.23)	*.011	.088	3.00 (0.42 – 21.38)	.190	.512
Forceps Minor	2.67 (0.43 – 16.61)	.265	.292	0.56 (0.10 – 3.24)	.400	.577
Inferior Fronto Occipital Fasciculus	7.41 (1.20 – 45.58)	.033	.088	1.62 (0.07 – 39.94)	.690	.759
Inferior Longitudinal Fasciculus	5.61 (1.07 – 29.57)	*.043	.088	2.24 (0.14 – 35.68)	.455	.577
Superior Longitudinal Fasciculus	4.56 (0.88 – 23.65)	.068	.104	1.93 (0.11 – 32.76)	.546	.648
Uncinate Fasciculus	3.80 (0.49 – 29.30)	.181	.206	0.76 (0.07 – 7.86)	.751	.788

Table S3

Influence of Interaction Terms of Gender and White Matter on Hazard Ratios for Transition to Psychosis Risk

Tract	Baseline Model			Longitudinal Model		
	HR (95% CI)	*p*	p_{corr}	HR (95% CI)	*p*	p_{corr}
Global FA	0.88 (0.32 – 2.41)	.781	.889	0.37 (0.04 – 3.60)	.304	.359
Anterior Thalamic Radiation	0.95 (0.36 – 2.48)	.902	.902	0.57 (0.08 – 4.16)	.495	.511
Cingulum Cingulate Gyrus	0.80 (0.29 – 2.20)	.636	.807	0.51 (0.14 – 1.83)	.226	.324
Cingulum Hippocampus	0.83 (0.26 – 2.71)	.745	.878	0.13 (0.00 – 4.47)	.193	.317
Corticospinal Tract	1.06 (0.40 – 2.79)	.900	.902	0.99 (0.31 – 3.20)	.980	.980
Forceps Major	1.30 (0.49 – 3.48)	.571	.797	0.28 (0.03 – 2.67)	.201	.317
Forceps Minor	0.79 (0.30 – 2.12)	.619	.807	0.47 (0.10 – 2.27)	.265	.339
Inferior Fronto Occipital Fasciculus	0.89 (0.33 – 2.44)	.810	.891	0.31 (0.03 – 3.80)	.277	.339
Inferior Longitudinal Fasciculus	0.81 (0.28 – 2.29)	.663	.810	0.20 (0.01 – 3.43)	.202	.317
Superior Longitudinal Fasciculus	0.76 (0.28 – 2.07)	.561	.797	0.24 (0.03 – 2.05)	.144	.317
Uncinate Fasciculus	0.93 (0.35 – 2.48)	.873	.902	0.43 (0.05 – 3.39)	.337	.370

Table S4

Hazard Ratios Consistently <1 Across All White Matter Tracts without Censoring at 3 Years

Tract	Baseline Model			Longitudinal Model		
	HR (95% CI)	*p*	p_{corr}	HR (95% CI)	*p*	p_{corr}
Global FA	0.58 (0.36 – 0.92)	*.024	.082	0.49 (0.23 – 1.02)	.055	.116
Anterior Thalamic Radiation	0.65 (0.41 – 1.01)	.054	.082	0.46 (0.23 – 0.92)	*.033	.116
Cingulum Cingulate Gyrus	0.66 (0.42 – 1.06)	.080	.087	0.63 (0.33 – 1.20)	.133	.146
Cingulum Hippocampus	0.44 (0.25 – 0.78)	*.007	.079	0.55 (0.23 – 1.33)	.151	.151
Corticospinal Tract	0.64 (0.40 – 1.02)	.059	.082	0.57 (0.29 – 1.10)	.081	.116
Forceps Major	0.61 (0.39 – 0.96)	*.034	.082	0.50 (0.27 – 0.95)	*.037	.116
Forceps Minor	0.70 (0.45 – 1.10)	.116	.116	0.51 (0.28 – 0.94)	*.036	.116
Inferior Fronto Occipital Fasciculus	0.63 (0.40 – 0.98)	*.043	.082	0.51 (0.26 – 0.99)	*.048	.116
Inferior Longitudinal Fasciculus	0.58 (0.37 – 0.93)	*.026	.082	0.52 (0.24 – 1.13)	.085	.116
Superior Longitudinal Fasciculus	0.63 (0.40 – 0.99)	*.046	.082	0.58 (0.29 – 1.16)	.103	.126
Uncinate Fasciculus	0.68 (0.43 – 1.05)	.079	.087	0.54 (0.27 – 1.07)	.070	.116

Figure S1

Baseline and Longitudinal Cox Proportional Hazards Models

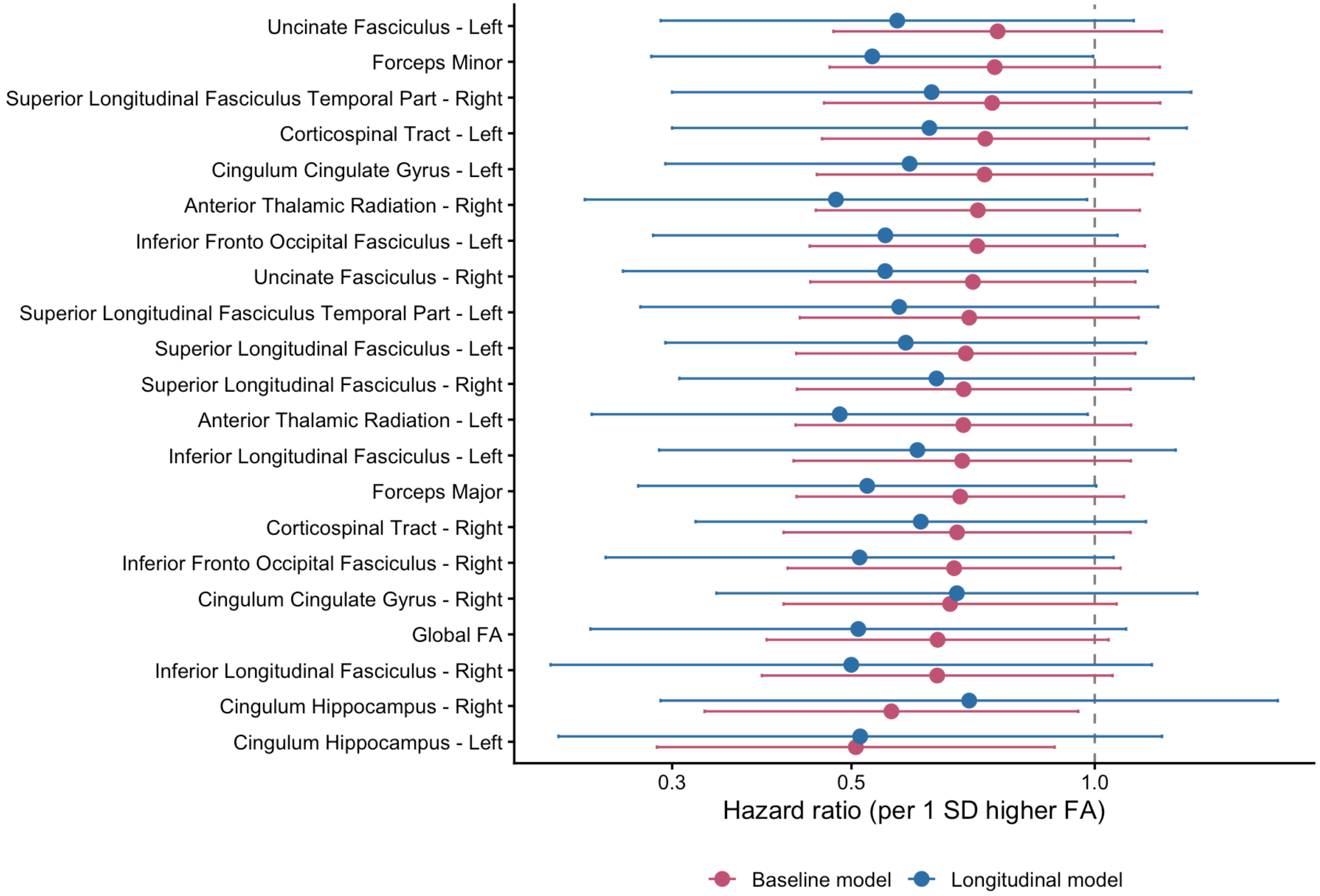

Supplement: Supplementary file 1 [file media-1.pdf]
